# Supplementary material for: Identifying the Active Sites of Heteroatom Graphene as a Conductive Membrane for the Electrochemical Filtration of Organic Contaminants
Source: Int J Mol Sci. 2022 Nov 29;23(23):14967. doi: 10.3390/ijms232314967 (PMC9739727; doi:10.3390/ijms232314967)
Supplement: Supplementary file 1 [file ijms-23-14967-s001.zip › ijms-2059740-supplementary.pdf]

Identifying the active sites of heteroatom graphene as a conductive membrane for the electrochemical filtration of organic contaminants

Meilan Pan, Junjian Li, Bingjun Pan*

College of Environment, Zhejiang University of Technology, Hangzhou, Zhejiang 310014, China

* To whom correspondence should be addressed (BJ Pan); E-mail: bjpan@zjut.edu.cn
Tel: +86 571 8881 3767
Fax: +86 571 8881 3767

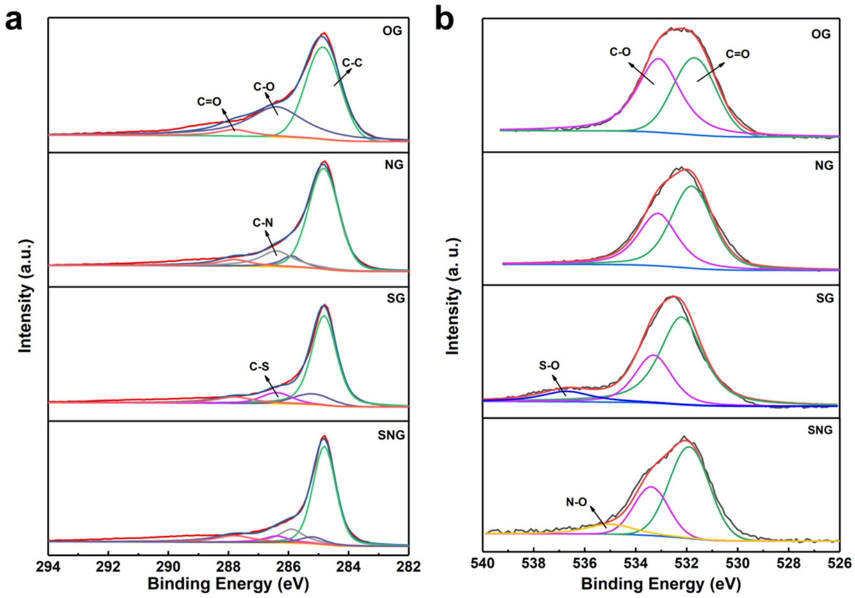

Figure S1. XPS spectra of C 1s (a) and O 1s (b).

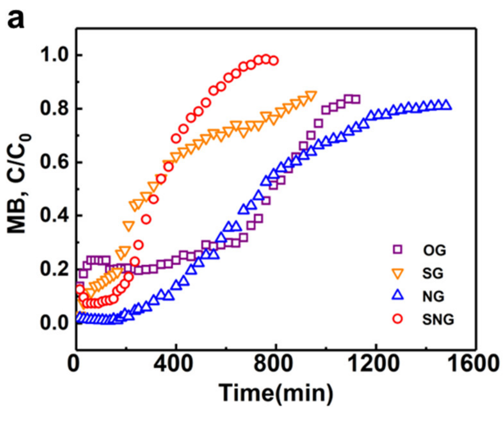

Figure S2. The adsorption curves of OG, NG, SG, and SNG for MB (a) and RhB (b).

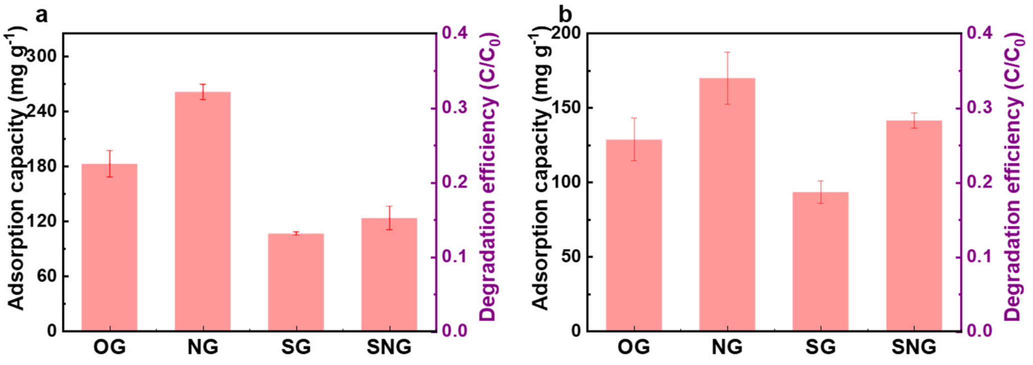

Figure S3. The adsorption capacity of OG, NG, SG, and SNG for MB (a) and RhB (b).

Table S1. The oxidation peak potentials (E_{pa}), reduction peak potentials (E_{pc}), and peak-to-peak potentials (ΔE_p) in the {$[Fe(CN)_6]^{4-/3-}$} and $Fe^{2+/3+}(H_2O)$ solution

Samples	**$[Fe(CN)_6]^{3-/4-}$**			**$Fe^{2+/3+}(H_2O)$**		
	E_{pa}, V	E_{pc}, V	ΔE_p, mV	E_{pa}, V	E_{pc}, V	ΔE_p, mV
NG	0.132	0.271	139	0.226	0.578	352
SG	0.138	0.265	127	0.362	0.470	108
SNG	0.123	0.289	166	0.169	0.676	507

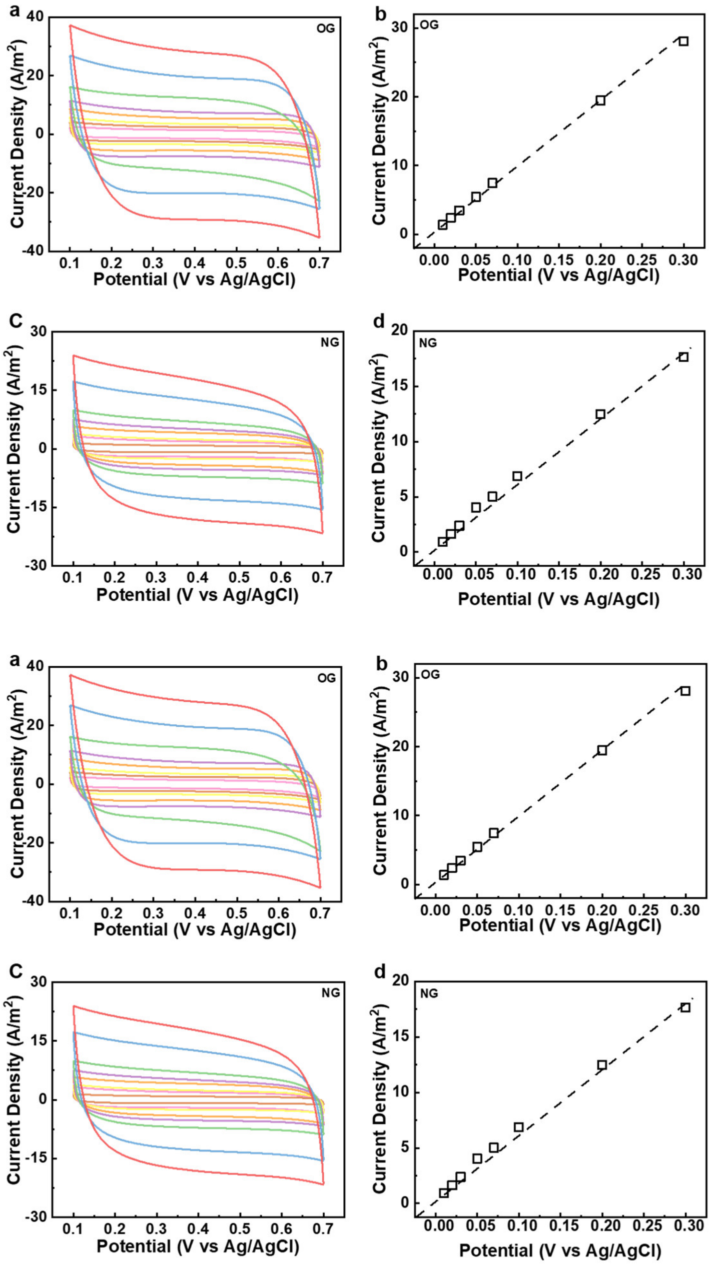

Figure S4. (a, c, e, g) CVs of OG, NG, SG and SNG collected in 0.1 M PBS solution and at different scan rates; (b, d, f, h) the corresponding current densities at 0.55 V (vs Ag/AgCl) as a function of scan rate.

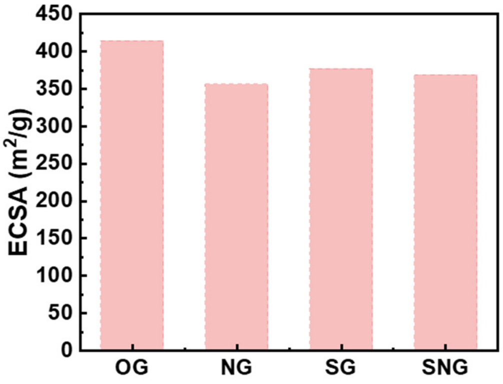

Figure S5. The ECSA of OG, NG, SG, and SNG.
